# Favorable effect of herbal extract on androgenic alopecia: A case report

**DOI:** 10.1097/MD.0000000000034524

**Published:** 2023-09-29

**Authors:** Yuxin Qian, Lijian Zhu, Lan Wu, Jingya Chen, Bin Ding, Yuanyuan Li, Yi Cao

**Affiliations:** a Zhejiang Chinese Medical University, Hangzhou, People’s Republic of China; b Department of Dermatology, The First Affiliated Hospital of Zhejiang Chinese Medical University, Hangzhou, People’s Republic of China; c The First Affiliated Hospital of Zhejiang Chinese Medical University, Hangzhou, People’s Republic of China.

**Keywords:** androgenetic alopecia, hair growth, traditional Chinese medicine, trichoscopy

## Abstract

**Rationale:**

Androgenic alopecia (AGA) is a prevalent condition with progressive miniaturization of hair follicles. Currently, reliable treatments have remained limited, and complementary medications for AGA are still being investigated. Traditional Chinese medicine formulas have conspicuous advantages in the treatment of AGA with good development prospects. Zimmer aqueous spray (ZAS) is a water spray containing Zimmer herbal extract powder (ZMWP), which consists of *Ligustri lucidi Fructus, Ecliptae Herba, Fallopia multiflora (Thunb.) Harald.* and *Polygonatum sibiricum Delar. ex Redoute*, etc. ZMWP is an active ingredient in the prevention of hair loss. Our aim is to provide evidence for the effectiveness of ZAS in the treatment of AGA.

**Patient concerns:**

A 41-year-old man had suffered from hair loss for 8 years.

**Interventions:**

The patient with moderate AGA received 3 to 4 mL ZAS daily or every other day for 3 months.

**Outcomes:**

The hair density obviously increased after 3 months of therapy. The improvement of hair diameter, vellus hair rate, and 1 hair pilosebaceous unit rate were observed with a trichoscopy and quantitatively analyzed. Besides, honeycomb pigment pattern mitigated and arborized red lines.

**Lessons:**

The results suggested that ZMWP might have the capability of improving hair growth and attenuating AGA, which can be a promising alternative treatment of AGA.

## 1. Introduction

Androgenic alopecia (AGA) is the most common type of progressive hair loss, which features miniaturization of the hair follicle and vellus transformation of terminal hair with a pattern distribution.^[1–3]^ AGA affects not only the hair condition, but the quality of life and self-esteem of patients. Although AGA is a prevalent problem, approved medications are still limited. Minoxidil^[4]^ and finasteride^[5]^ are the 2 popular medications for AGA treatment, which have been approved by US Food and Drug Administration. The combination of topical minoxidil and finasteride is an approved therapeutic option with over 90% effectiveness after 6 months treatment.^[6]^ Subsequently, the adverse effects of minoxidil and finasteride including hypertrichosis, lightheadedness and other systematic effects have been reported for decades.^[7]^ Besides, the minoxidil and finasteride application should be life-long to keep the great efficacy. Hence, most AGA patients discontinue the aggressive treatment, and choose simplified methods, such as using anti-thinning shampoos. Even, the anti-AGA efficacy of some shampoos is uncommendable. Therefore, novel complementary and alternative medicines for AGA amelioration need to be investigated.

A growing number of studies indicated that herbal alternatives have favorable effects on alopecia.^[8]^ Zimmer herbal extract powder (ZMWP) is an herbal formula based on Chinese traditional medicine “erzhi” pill, which has been proved with the effects of hair loss attenuation, hair growth improvement.^[9]^ According to the theory of Traditional Chinese medicine, the deficiency on the kidney and liver is the basic pathogeny of AGA.^[10]^ And “erzhi” pill is an herbal prescription, which benefits the health of the kidney and liver.^[11]^ *Ecliptae Herba* and *Platycladi Cacumen*, the main herbals of ZMWP, can regulate steroid 5-α-reductase^[12]^ which is an effective therapeutic target for AGA. Extracts of *Platycladi Cacumen* could promote the proliferation of dermal papilla cells.^[13]^ *Polygonum multiflorum* could reverse the androgenic effects of dihydrotestosterone and prolong the anagen of human hair follicles.^[14]^ We used to apply the ZMWP as an active fraction of anti-AGA shampoo. And it is the first time, we prepared ZMWP as an aqueous spray, Zimmer aqueous spray (ZAS), and evaluated the AGA ameliorative effects.

In this study, a male adult, who suffered from progressive hair loss and had been diagnosed as AGA, applied ZAS for 3 months. And an obvious hair growth promoting efficacy of the spray could be observed with trichoscopy.

## 2. Methods

### 2.1. Patient

A 41-year-old man had suffered from alopecia for almost 8 years, with receding hairline and hair loss. Diagnosis of AGA was based on detailed medical history, clinical examination and laboratory tests. Laboratory tests included:

1. CBC, CRP.
2. Serum ferritin, Vitamin B12.
3. Folic acid.
4. T3, T4, TSH, FT3, FT4, anti-TPO, Tg, TGAb, TRAb.
5. Rheumatoid factor.
6. FSH, LH, Progesterone, E2, PRL, Androgen.

Laboratory tests were performed to exclude other hair loss causes, such as anemia, poor nutrition, thyroid dysfunction or discoid lupus erythematosus. The laboratory examinations of him were all normal and he was presented with oily scalp in otherwise excellent health. Besides, he was a positive family history of alopecia which is inherited from the father. Combined with medical history and family history, he was diagnosed with AGA. Before the treatment, he had received no other medical therapy but anti-dandruff shampoo.

### 2.2. Preparation of ZAS and treatment

We dissolved 4 g ZMWP with 120 mL distilled water, sterile filtrated and aliquoted into an aseptic spray bottle, which has been kept in 4°C. In 3 months, the volunteer applied once daily or every other day 3 to 4 mL ZAS after shower. Otherwise, the volunteer used ordinary anti-dandruff shampoo for head and shoulders as usual during the therapeutic period.

Our study was approved by the ethical committee (2022-KLS-229-02). The study protocol complied with the Declaration of Helsinki. Informed consent was obtained from the patient for publication of this case report details

### 2.3. Trichoscopy

Using trichoscopy (BN-YQTC-1001, Nanjing Beining medical equipment Co. Ltd, Nanjing, China) to observe and record the condition of scalp. Trichoscopy was conducted before treatment and at 3 months after the first treatment.

### 2.4. Statistical analysis

Data are informed as mean ± SD. Statistical analysis was prepared in IBM SPSS Statistics for Windows (Version 26.0, IBM Corp, Armonk, NY) and Microsoft Excel 2021. Differences between the groups were computed by Student *t* test or rank sum test. *P* value < .05 was considered statistically significant for all tests.

## 3. Results

### 3.1. Increasing hair density

**Figure 1.**
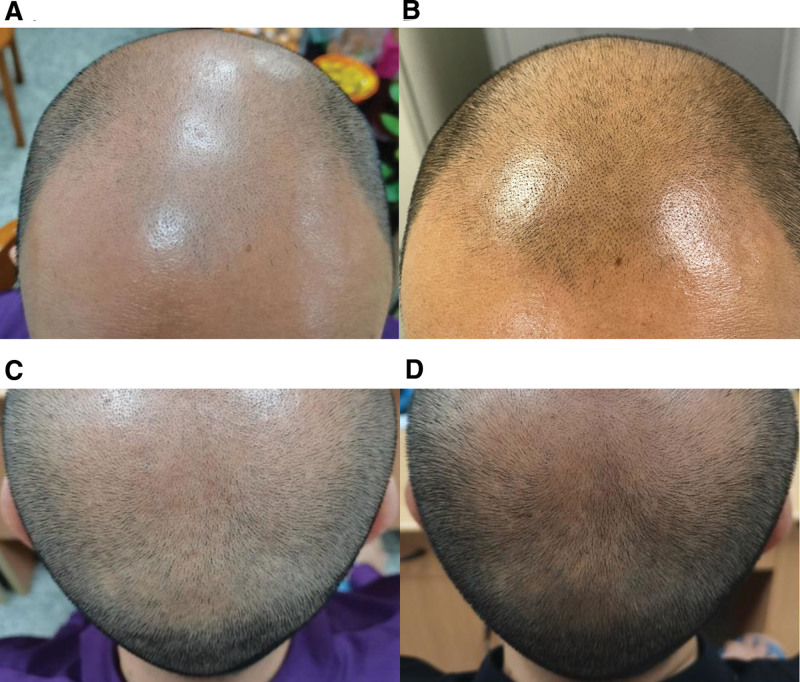
Photographs of patient’s scalp: (A) The frontal scalp before treatment. (B) The frontal scalp after 3 months treatment. (C) The scalp of the top of the head before treatment. (D) The scalp of the top of the head after 3 months treatment.

**Figure 2.**
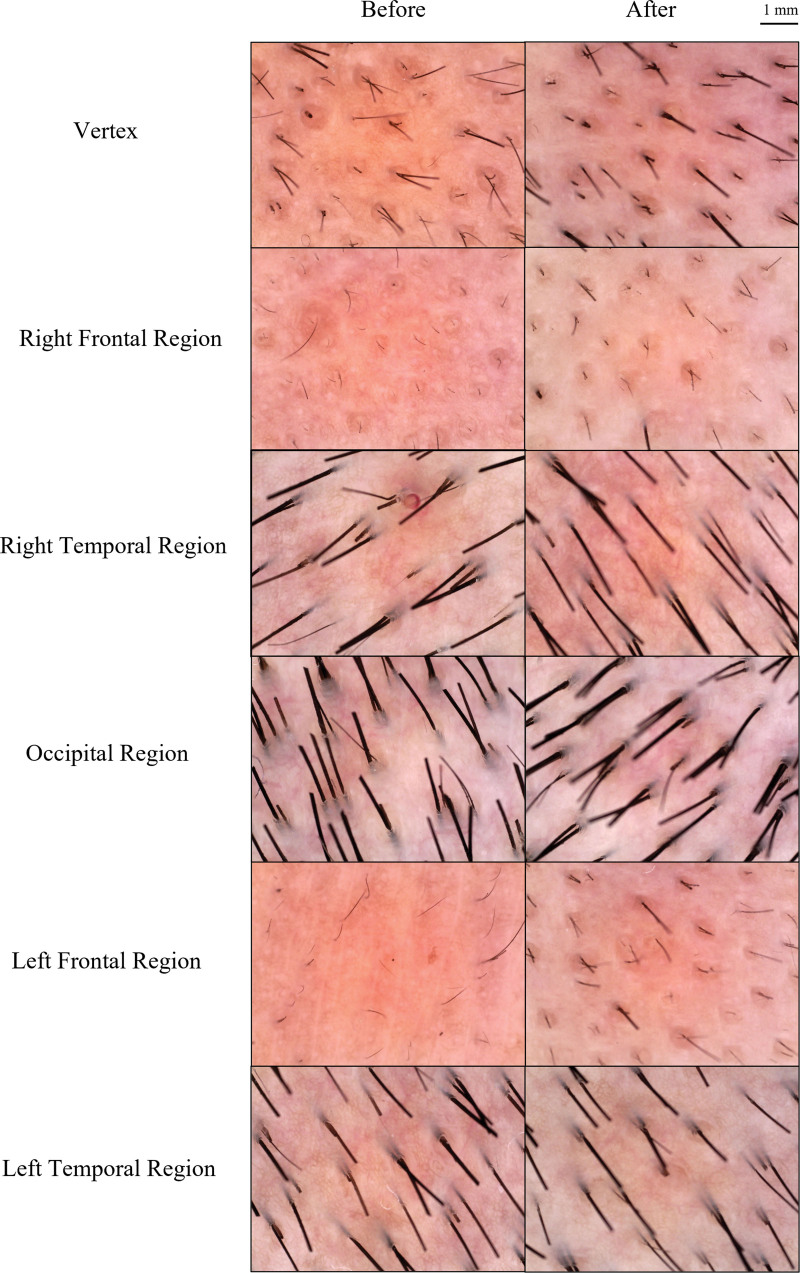
The hair and scalp condition before or after treatment under the tricoscope. Hair density and quantity improved, especially at vertex, left frontal region and right frontal region. Honeycomb pigment pattern and arborizing red lines relieved.

Before the treatment, a noticeable hairless was observed, which was extremely severe on the vertex and frontal region. On appearance, his front hairline was extremely receding. There was almost no hair at the frontal region and vertex, while the occipital hair was relatively thick and dense (Fig. 1A and C). Under trichoscopy, apparent miniaturization of the hair follicle had been observed. The frontal region and vertex were dominated by empty follicles with only several short vellus (Fig. 2).

Since the 4th week, the volunteer described the increased strength of hair fibers. He complained the trouble of using Liquid spray and did not report any adverse effect. We observed a significantly increased hair density in 3 months. Bare scalp area reduced and obvious hair growth was observed, especially at vertex and frontal region (Fig. 1B and D). Trichoscopic photographs showed an overall improvement in hair density and quality, as his hair became thicker, normal hair (Fig. 2).

### 3.2. Increasing hair diameter

As shown in Figure 2, the hair density and diameter increased significantly in the patient (Fig. 2). Detailly, the mean hair diameter of the vertex was 45.62 μm, right frontal hair was 31.55 μm and right frontal hair was 41.75 μm. It was significantly improved in comparison with pretherapy, which were 33.46 mm, 24.48 mm and 21.83 mm respectively (*P* < .001, *P* = .03 and *P* < .001) (Table 1). The improved thickness of hair on right temporal, occipital and left temporal hair were also observed. The diameter of which increased from 80.25 mm to 82.38 mm, 80.37 mm to 97.07 mm, and 74.93 mm to 84.70 mm respectively.

**Table 1:** The hair diameter, percentage of vellus hair and percentage of 1 hair pilosebaceous unit of different region before or after treatment. *P < .05, **P < .01, ***P < .005, ****P < .001.

Area of head	Average hair diameter (um)		*P* value	Vellus hair (%)		1 hair pilosebaceous unit (%)	
	Before	After		Before	After	Before	After
Vertex	33.46 ± 13.65	45.62 ± 13.44	<.001	/	/	0.1228	0.2750
Right Frontal Region	24.48 ± 10.04	31.55 ± 13.59	.034	0.7931	0.6053	0.5238	0.2105
Right Temporal Region	80.25 ± 21.75	82.38 ± 14.07	.491	/	/	0.1667	0.1563
Occipital region	80.37 ± 22.05	97.07 ± 36.57	.094	/	/	0.2667	0.1714
Left frontal region	21.83 ± 7.48	41.75 ± 11.33	<.001	0.8611	0.5000	0.6111	0.4211
Left temporal region	74.93 ± 16.60	84.70 ± 18.22	.053	/	/	0.3103	0.2333

### 3.3. Improvement of scalp and hair follicle condition

The amelioration of scalp condition was observed with trichoscopy. Honeycomb pigment pattern mitigated and arborizing red lines relieved (Fig. 2). One character of the AGA is the increased number of vellus hairs instead of healthy terminal hair.^[1]^ Hence, the percentage of vellus hair on left and right frontal sections were evaluated in this study. And 50.00% and 60.53% vellus hair was at left and right frontal regions after 3 months treatment, which were 86.11%, 79.31% initially (Table 1). Besides, the percentage of 1 hair pilosebaceous unit decreased. Especially at the frontal region, it decreased from 61.11% to 42.11%, and 52.38% to 21.05% at left and right side, respectively.

## 4. Discussion

This project is to evaluate the benefic effect of ZMWP on AGA treatment. ZMWP is the aqueous extract of an herbal prescription. Its anti-hair loss activity has been demonstrated with some cell and animal experiments. Additionally, some of volunteers, who suffered from hair loss, reported the benefit effects after using of the shampoo containing ZMWP for months. All of these results were used for patent application (still in verification). However, the anti-AGA capability of ZMWP is still not declared.

In this study, we prepared the ZMWP as ZAS to eliminate the effects of shampoo. And we observed the favorable effect of ZAS on AGA (Fig. 1). In detail, the increased hair diameter, decreased vellus hair and eliminated the 1 hair pilosebaceous unit had been contrastively quantified before and after the ZAS treatment (Table 1). Otherwise, we also found the improvement of scalp condition with the help of trichoscopy, such as the lighted of the skin-tone, mitigated honeycomb pigment pattern and relieved arborizing red lines (Fig. 2).

Progressive thinning of hair was a feature of AGA.^[15]^ It has been reported that hair diameter reduced by about 1 micron per year in males with AGA.^[16]^ Vellus hair is a kind of tinny hair with a diameter under 30 μm, some of which can generally develop into terminal hair. However, the increased percentage of vellus hair indicated the miniaturization of hair follicles.^[17]^ Therefore, the increased hair diameter and decline of vellus hair ratio represented the health of hair follicles. ZAS treatment could increase the mean hair diameter although the improvement in some regions were not statistically significant. It might depend on the health status of the hair follicles. Scalp honeycomb pigment and the arborizing red lines (capillary vessels) were recognized as superficial dermal telangiectasias and were correlated to severity of AGA.^[18]^ As well as, the scalp 1 hair follicular unit ratio reflect severe AGA.^[19]^ Our trichoscopy findings illustrated the ameliorative effect of ZAS on AGA.

The classical treatments of AGA were Finasteride 1 mg or/and minoxidil 2% to 5 % solution. A random trial conducted by Ghonemy et al^[20]^ using of 5% topical minoxidil twice a day on 36-weeks reported that mean of hair density per square centimeter significantly increased 0.74 at vertex scale and 0.59 at frontal scale, and the mean hair thickness increased 0.94 μm and 1.35 μm at vertex and frontal scale respectively. However, the side effect of minoxidil on hypertrichosis and increasement in pulse rate limited its therapeutic application.^[21]^ In our case, the mean hair thickness under ZAS treatment increased 19.92 μm, 7.07 μm at left and right frontal region, and 12.16 μm at vertex, which seemed better than 5% minoxidil treatment in Ghonemy study. However, it has been known that various factors, such as nationality, age, nutrition, stress, could influence the development of AGA.^[1]^ Owing to the fact that only 1 volunteer was engaged in this case study, the study invited more volunteers is still needed.

Herbal recipes for AGA treatment have been investigated for decades. A randomized controlled study demonstrated the effect of a topical herbal solution which includes *Rosmarinus officinalis Linn, Olea europaea L*., and lipidosterolic extract of *Serenoa repens.*^[22]^ In this study, the mean of hair diameter of 5% minoxidil combined with the topical herbal solution treatment group were 60.92 μm and 62.67 μm at 24 and 36-week which were significantly thicker than that of the group with only 5% minoxidil topical solution treatment (51.17 μm and 50.58 μm). This study suggested that herbal potion treatment could improve the effect of minoxidil. This reminded us that ZAS application might increase the efficiency of minoxidil or finasteride, as well as ameliorate the harmful effects. Besides, in this case, the main component of *Olea europaea L.* is Oleuropein which is also the constituent of ZMWP. In fact, nowadays, it is still a challenging object to elucidate the chemical compounds constitute and the involved scientific mechanism of an herbal potion. But the scientists have never given up on sufficiently declaring the scientific cognition of the effective substances and mechanism of Chinese medicine.

Besides herbal remedies, many other complementary therapeutic options have also been developing. Scalp micro needling was reported as an effective way via releasing platelet-derived growth factor, activating follicle stem cells and upregulating the expressing hair growth-related genes.^[24]^ In 2015, Dhurat et al^[23]^ reported a case series of 4 men who showed no new hair growth under minoxidil treatment.^[24]^ The study showed that microneedling along with minoxidil treated group has better statistical results compared to minoxidil treated group in promoting hair growth in men with AGA.^[23]^ These enlighten us that the combination of microneedling with ZAS treatment could also be taken into consideration.

## 5. Conclusion

While AGA is recognized as the most common type of progressive hair loss, medicine choices are still limited and with obvious side effects. In this case, a 41-year-old man with stage VII AGA showed an excellent recovery after ZAS treatment for 3 months. Our case study indicated the anti-AGA activity of ZMWP and the therapeutic application possibility of ZAS on AGA treatment. Although our case study provides an alternative treatment of AGA, there are some still left unknown, such as the possible side effects, the active ingredients and the involved pharmacological mechanisms, which still remains for further researching.

## Acknowledgments

The authors thank Ms. Ting Zhang, College of Life Science, Zhejiang Chinses Medical University, for her greatly helping on volunteer recruitment.

## Author contributions

**Conceptualization:** Yuxin Qian, Lijian Zhu, Yi Cao.

**Data curation:** Yuxin Qian, Lan Wu, Yuanyuan Li.

**Formal analysis:** Yuxin Qian, Lijian Zhu.

**Investigation:** Yuxin Qian, Lijian Zhu, Lan Wu, Jingya Chen.

**Methodology:** Lan Wu.

**Project administration:** Lan Wu.

**Resources:** Yuxin Qian, Lan Wu, Jingya Chen, Bin Ding, Yuanyuan Li.

**Software:** Yuxin Qian, Lijian Zhu.

**Supervision:** Lan Wu, Bin Ding.

**Validation:** Yuxin Qian, Lijian Zhu, Lan Wu, Jingya Chen, Bin Ding.

**Visualization:** Yuxin Qian, Lijian Zhu.

**Writing – original draft:** Yuxin Qian.

**Writing – review & editing:** Lijian Zhu, Bin Ding, Yi Cao.
